# Supplementary material for: From sequence to enzyme mechanism using multi-label machine learning
Source: BMC Bioinformatics. 2014 May 19;15:150. doi: 10.1186/1471-2105-15-150 (PMC4229970; doi:10.1186/1471-2105-15-150)
Supplement: Additional file 2 — Java code of ml2db. Additional file ml2db_code.tar.gz contains the Java source code to run the multi-label machine learning experiments and save the results to database. The code’s Javadoc is included. [file 1471-2105-15-150-S2.zip › additional file 2/ml2db/ml2db/doc/test/ml2db/al/utility/confidence/ConfidenceCalculatorTest.html]

ConfidenceCalculatorTest


JavaScript is disabled on your browser.


- Overview
- Package
- Class
- Use
- Tree
- Deprecated
- Index
- Help

- Prev Class
- Next Class

- Frames
- No Frames

- All Classes

- Summary:
- Nested |
- Field |
- Constr |
- Method

- Detail:
- Field |
- Constr |
- Method


test.ml2db.al.utility.confidence

## Class ConfidenceCalculatorTest

- java.lang.Object
- - junit.framework.Assert
  - - junit.framework.TestCase
    - - test.ml2db.al.utility.confidence.ConfidenceCalculatorTest

- All Implemented Interfaces:
  :   junit.framework.Test

  ---

    

  ```
  public class ConfidenceCalculatorTest
  extends junit.framework.TestCase
  ```

- - ### Constructor Summary

    Constructors

    Constructor and Description

    `ConfidenceCalculatorTest()`
  - ### Method Summary

    Methods

    | Modifier and Type | Method and Description |
    | --- | --- |
    | `static ConfidenceCalculator` | `getConfidenceCalculator()` |
    | `static IdMultiLabelInstances` | `getInstancesOfTrainedLerner1()` |
    | `static IdMultiLabelInstances` | `getInstancesOfTrainedLerner2()` |
    | `static mulan.classifier.MultiLabelLearner` | `getTrainedLearner(IdMultiLabelInstances instances)` |
    | `static mulan.classifier.MultiLabelLearner` | `getTrainedLearner1()` |
    | `static mulan.classifier.MultiLabelLearner` | `getTrainedLearner2()` |
    | `void` | `testGetAverageConfidenceUncertainty()` |
    | `void` | `testGetAverageUtility()` |
    | `void` | `testGetConfidences()` |
    | `void` | `testGetUncertainty()` |

    - ### Methods inherited from class junit.framework.TestCase

      `countTestCases, getName, run, run, runBare, setName, toString`
    - ### Methods inherited from class junit.framework.Assert

      `assertEquals, assertEquals, assertEquals, assertEquals, assertEquals, assertEquals, assertEquals, assertEquals, assertEquals, assertEquals, assertEquals, assertEquals, assertEquals, assertEquals, assertEquals, assertEquals, assertEquals, assertEquals, assertEquals, assertEquals, assertFalse, assertFalse, assertNotNull, assertNotNull, assertNotSame, assertNotSame, assertNull, assertNull, assertSame, assertSame, assertTrue, assertTrue, fail, fail, failNotEquals, failNotSame, failSame, format`
    - ### Methods inherited from class java.lang.Object

      `equals, getClass, hashCode, notify, notifyAll, wait, wait, wait`

- - ### Constructor Detail


    - #### ConfidenceCalculatorTest

      ```
      public ConfidenceCalculatorTest()
      ```
  - ### Method Detail


    - #### testGetAverageConfidenceUncertainty

      ```
      public void testGetAverageConfidenceUncertainty()
      ```


    - #### testGetAverageUtility

      ```
      public void testGetAverageUtility()
      ```


    - #### testGetConfidences

      ```
      public void testGetConfidences()
      ```


    - #### testGetUncertainty

      ```
      public void testGetUncertainty()
      ```


    - #### getConfidenceCalculator

      ```
      public static ConfidenceCalculator getConfidenceCalculator()
      ```


    - #### getInstancesOfTrainedLerner1

      ```
      public static IdMultiLabelInstances getInstancesOfTrainedLerner1()
      ```


    - #### getInstancesOfTrainedLerner2

      ```
      public static IdMultiLabelInstances getInstancesOfTrainedLerner2()
      ```


    - #### getTrainedLearner

      ```
      public static mulan.classifier.MultiLabelLearner getTrainedLearner(IdMultiLabelInstances instances)
      ```


    - #### getTrainedLearner1

      ```
      public static mulan.classifier.MultiLabelLearner getTrainedLearner1()
      ```


    - #### getTrainedLearner2

      ```
      public static mulan.classifier.MultiLabelLearner getTrainedLearner2()
      ```


- Overview
- Package
- Class
- Use
- Tree
- Deprecated
- Index
- Help

- Prev Class
- Next Class

- Frames
- No Frames

- All Classes

- Summary:
- Nested |
- Field |
- Constr |
- Method

- Detail:
- Field |
- Constr |
- Method
